# Letter to the editor: reply to Destaillats, interesterified fats to replace trans fat

**DOI:** 10.1186/1743-7075-4-13

**Published:** 2007-05-14

**Authors:** Kalyana Sundram, Tilakavati Karupaiah, KC Hayes

**Affiliations:** 1 Food Technology & Nutrition Research Unit, Malaysian Palm Oil Board, Kuala Lumpur, Malaysia; 2 Faculty of Allied Health Sciences, National University of Malaysia, Malaysia; 3 Foster Biomedical Research Lab, Brandeis University, Waltham, MA, USA

## Abstract

Although more sophisticated ways exist to analyze TG-MS than that applied in our study, the approach was able to identify the TG species sufficiently to emphasize the importance of TG structure. The criticism that differences in dietary fat saturation alone would explain the lipoprotein response across diets is not supported by careful scrutiny of the facts. Nor does fat saturation per se address the observed impact that fat structure had on insulin/glucose metabolism.

Title. Reply to Destaillats: Interesterified fats to replace trans fat.

Dear Editor,

The letter from Destaillats et al [1] takes issue with the design of our recent study [2], suggesting that we failed to run a true test of fatty acid exchanges that would account for differences in fat saturation between the three fats tested. In addition they suggest that our analysis of TG-MS may lack rigor. If our only objective was simply to match fatty acid exchanges to generate fats with identical melt points, their argument concerning design would be well taken. But, in fact, our intention was broader in scope as we wished to examine three types of fat products (a natural saturated fat, POL; one enriched with TFA, PHSO; and an interesterified fat, IE, enriched with 18:0) that are used in certain product formulations where a relatively high degree of solid fat is required. These three fats contained rough approximations for both total SFA+TFA (12–18%en) and PUFA (3.5–7 %en). Not only was the FA exchange of interest, but the structure of TG was a consideration, as well, because both partial hydrogenation and IE processing modify fat molecular structure. The critique [1] essentially ignores the TG structure issue, even though structure repeatedly surfaces as the most logical explanation for results observed.

On the matter of TG-MS analysis, they are correct to indicate that pancreatic lipase, Grignard reagent, mass-spectrometry, or NMR have been utilized successfully in the past. However, our reported HPLC coupled to a ELSD (Evaporative Light Scatter Detector) method allowed us to detect the major TG molecules reported, whereas mass spec would be more appropriate when separation of two TG regio-isomers is essential. Despite this limitation, our observations and conclusion about the possible influence of TG structure on plasma lipoproteins, glucose and insulin are not invalidated. Our cited technique is commonly used by industry to generate basic TG isomer profiles when assessing functionality of fats. It allows adequate discrimination of TG species provided peaks are matched with authentic TG-MS standards. In continuing analysis, plasma TG samples from this study were analyzed for their sn-2 fatty acid content using pancreatic lipase, TLC separation, and GC-fatty acid analysis. A striking and significant increase in the sn-2 stearic acid was observed following consumption of the IE fat compared to both other dietary treatments (unpublished), confirming our point that formation of TG-MS with sn2 stearic acid during preparation of IE fat carried over to the metabolism of this fat.

Their additional point that differences in total saturated fatty acids between diets would explain differences in LDL and HDL does not adequately account for our observations for several reasons. First, although IE had more total SFA than POL or PHSO, most of this was as 18:0, which is generally deemed to be neutral, or even cholesterol lowering, if we accept their added reference [3] as part of the argument. If 18:0 is neutral or cholesterol-lowering, then it seems illogical to include it among the SFA, which they suggest is the reason that LDL was higher during IE fat intake, ie. because IE contained more SFA than POL. Palmitic acid, which has been described as the most cholesterol-raising [3], was much greater in our POL diet, yet produced the lowest LDL and highest HDL. They also acknowledge that the Mensink et al regression analysis [4] failed to account for the lower HDL level during IE consumption. But Thijessen and Mensink [3] specifically point out that in relation to IE fats "..it is possible that the effects of natural fats rich in stearic acid on the serum lipoprotein profile are different from those of synthetic fats". Thus, the application of regression analysis is suspect at best under conditions of IE fat consumption. Furthermore, the IE diet had twice the amount of PUFA as the POL diet, which by most counts and regression analyses would clearly favor LDL-cholesterol lowering by IE [5]. So if IE and TFA begin to look alike in their metabolic outcomes yet differ from POL, and if said differences cannot be traced to fatty acid saturation/unsaturation, it seems most likely that interesterification with 18:0 and TG structure in general are problematic here, not fatty acid saturation. Our interpretation simply attempted to emphasize this point.

Finally and more importantly, the focus on lipoproteins and fat saturation misses the real message in our report, ie. modifying TG structure by IE or partial hydrogenation is seemingly able to alter glucose and insulin metabolism in a surprisingly short period relative to a natural saturated fat. The alteration may be most dramatic in the extreme, as observed with the IE fat fed here. Further evaluation seems warranted because IE fats continue to raise questions about atypical outcomes, whether it be when interpreting a regression analysis involving 18:0 and the lipoprotein response [3] or the plasma glucose/insulin outcomes reported in our study. We agree that it would be nice if others, such as the authors of the letter, were to test additional hypotheses involving IE, including that which they suggest. With enough studies it should be possible to determine at what level IE fats become a problem metabolically.
